# Gender and Facial Dominance in Gaze Cuing: Emotional Context Matters in the Eyes That We Follow

**DOI:** 10.1371/journal.pone.0059471

**Published:** 2013-04-03

**Authors:** Garian Ohlsen, Wieske van Zoest, Mark van Vugt

**Affiliations:** 1 Vrije Universiteit Amsterdam, Amsterdam, The Netherlands; 2 Center for Mind/Brain Sciences, University of Trento, Trento, Italy; 3 University of Oxford, Oxford, United Kingdom; University of Bologna, Italy

## Abstract

Gaze following is a socio-cognitive process that provides adaptive information about potential threats and opportunities in the individual’s environment. The aim of the present study was to investigate the potential interaction between emotional context and facial dominance in gaze following. We used the gaze cue task to induce attention to or away from the location of a target stimulus. In the experiment, the gaze cue either belonged to a (dominant looking) male face or a (non-dominant looking) female face. Critically, prior to the task, individuals were primed with pictures of threat or no threat to induce either a dangerous or safe environment. Findings revealed that the primed emotional context critically influenced the gaze cuing effect. While a gaze cue of the dominant male face influenced performance in both the threat and no-threat conditions, the gaze cue of the non-dominant female face only influenced performance in the no-threat condition. This research suggests an implicit, context-dependent follower bias, which carries implications for research on visual attention, social cognition, and leadership.

## Introduction

Eye gaze is a very powerful cue in numerous social species including humans. Humans rapidly shift their attention in the direction in which other individuals are looking [1]–[3]. Not surprising, the eyes are the area of the human face that people most quickly and frequently attend to [4], [5] and non-human primates possess neurons specifically designed to respond to eye gaze and head orientation [6]. It is thought that monitoring and following the eye gaze of others is an evolutionary adaptive process because it provides useful information about potential threats and opportunities in the organism’s environment [7]. In humans, gaze following play an important role in collaboration, coordination, social learning, threat assessment, status, dominance, and understanding the intentions of others [3], [7]–[11]. Cognitive scientists typically use a gaze cuing task to study the influence of gaze on attentional deployment. In such tasks a central face is presented whose diverted gaze is either directed toward the target stimulus (valid cue) or away from the target stimulus (invalid cue). The results typically show that participants respond faster, and with fewer errors, to a validly cued target than to an invalidly cued target. This is also known as the ‘gaze cuing effect’ [1], [2], [12]. Note that this effect occurs despite the fact that the gaze direction does not actually predict the location of the target stimulus.

Previous experimental research has demonstrated that cues associated with social status influence the gaze cuing effect. A study in primates shows that macaque monkeys follow the gaze of high status monkeys more than of low status monkeys especially at larger viewing times (>400 ms) [13]. Comparable results are found in human populations: People selectively attend more to individuals rated as high in social status, and they especially look at their eye regions [14]. Other research has shown that the gaze cuing effect is stronger when participants look at a face of an individual being described as high in status [15]. Social status may be conveyed through certain facial features. For example, evidence suggests that certain facial cues of dominance [16], [17] and competence [18], [19] determine who people selectively attend to and attribute leadership to. A recent experiment showed that the gaze cuing effect was enhanced by using masculinized face morphs but this effect decreased at larger viewing times (>400 ms) [9]. Facial masculinity and leadership are correlated in real-life [20] and so are facial masculinity and perceptions of dominance [21]. These findings suggest that it is adaptive for individuals to know the direction in which high status individuals or group leaders are looking [22].

Other contextual factors such as familiarity and group membership may also influence gaze cuing. A recent study [23] shows that familiarity increases the gaze cuing effect in women but not in men. In this work, participants were either familiar or unfamiliar with the faces presented as gaze cue. The results showed enhanced cuing effects only for the female participants when confronted with familiar faces. A related study [10] showed that racial group membership (white and black faces) modulates gaze cuing. White participants followed the gaze cues of white faces only, whereas black participants followed both the white and black face gaze cues. Similar effects were found in a study in which the amount of gaze cuing was measured among in-group and out-group voters when they were presented with left and right winged Italian political characters [24].

In sum, there is evidence that gaze cuing might be influenced by facial cues signaling status, dominance, leadership, familiarity and group membership. The aim of the present research is to extend this work by investigating whether a certain feature of the cued face interacts with a specific emotional context to affect gaze cuing. Informed by an evolutionary psychological perspective we hypothesize that it is evolutionarily adaptive for humans to follow dominant individuals especially in times of threat and danger, because they offer safety and protection. Research shows that people have a stronger preference for dominant looking, masculine leaders in war and non-dominant looking, feminine leaders in peace [16], [20], [25]. Extrapolating these findings, we predict that facial cues of dominance enhance gaze cuing only under conditions of emotional threat. Specifically, the present study investigates whether gaze cuing towards a dominant looking male or non-dominant looking female face differs as a function of exposure to threatening or non-threatening stimuli. A standard gaze cuing task was employed to investigate this hypothesis. In this task we presented either a dominant male face or a non-dominant female face as gaze cue. Critically, participants were primed with emotional pictures signifying either “threat” or “no threat” to induce either a dangerous or safe environment, respectively. We predict that the gaze cue effect will be stronger, overall, when participants are exposed to dominant male faces versus non-dominant female faces. Importantly, the difference in gaze cuing between these faces should be particularly pronounced after exposure to a threatening emotional context. For exploratory purposes, and consistent with previous studies, we include both short (200 ms) and longer viewing times (800 ms) for the facial cues in our design.

## Methods

### Participants

Forty-nine Dutch speaking participants (22 men, 27 women; Mean age = 23.82 years, SD = 7.87 years) participated in this study for money or course credits. Kolmogorov-Smirnov tests showed that all gaze-effect cuing scores were normally distributed in the different conditions (all *D*<.15 all *p*>.20). The participants were all tested under the same conditions. For all tasks written consent was obtained for all participants and the research was approved by the ethics committee from the Faculty.

### Apparatus and Materials

**Figure 1:**
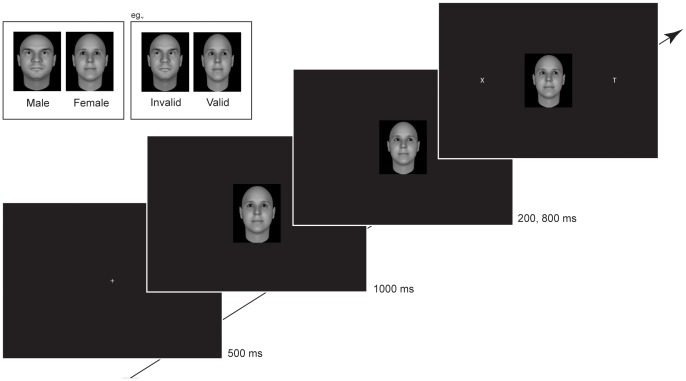
Experimental design. Top of the figure: The male (right) and female (left) face with a straight ahead gaze, a gaze to the right and a gaze to the left. Bottom of the figure: The used gaze cuing task that consists of a fixation point which was visible for 500 ms, a (male/female) face with a straight ahead gaze appears after the fixation point for 1000 ms, then the gaze of the (male/female) face looks to the left, right or straight ahead (control condition, no change) and the target letter ‘L’ or ‘T’ appears after 200 or 800 ms (SOA) and stays visible until the participants press the up (‘T’) or down (‘L’) arrow on the keyboard.

One male and one female face, starkly differing in facial dominance, were selected from the database of the *Social Cognition & Social Neuroscience Lab* [26] for which faces were created with the Facegen Modeller program (http://facegen.com [27]). The morphed dominant male face was selected from the dominance database and highly dominant (3 standard deviations more dominant than his neutral face). The female face was selected from the database with randomly generated faces and was rated as low in dominance (3.52 on a 9 points scale; n = 228). The female face was matched to the male face on the basis of skin color and overall face shape. The two directional gaze images (gaze left and right) were created from the straight-ahead gaze (see Figure 1) using Photoshop.

For the two prime conditions (threat/no threat), photographs were selected from the IAPS (International Affective Picture System) database for emotional pictures (No threat IAPS [2008] picture numbers: 1710, 1750, 2040, 2070, 2154, 2156, 2165, 2352.1, 2530, 2540, 2550, 4597, 4610, 4622, 5210, 5760, 5780, 5833, 8461, 8497; Threat IAPS [2008] picture numbers: 1304, 1930, 3064, 3120, 3500, 3530, 6230, 6260, 6312, 6560, 9040, 9075, 9332, 9410, 9421, 9429, 9630, 9908, 9930, 9940) [28]. The selection of threat versus no threat pictures was based on ratings of pleasure (low vs. high), dominance (high vs. low), and arousal (high vs. low), according to the standardized affective rating system SAM [29]. The twenty threat pictures included pictures that scored low on pleasure, high on dominance and arousal, and contained pictures with, for example, graphic displays of attacks, combat situations, and accidents. The twenty no threat pictures included pictures that rated high on pleasure, low on dominance and arousal, for example, smiling babies, couples holding hands, beautiful nature scenes, and cute animals. Each picture prime was presented for 3000 ms. There was no inter-stimulus-interval between the pictures. The presentation of a complete prime condition with twenty pictures took 1 minute to complete.

The stimuli were presented on a 15.6 inch iiYama HM903DT screen with a resolution of 1024×768 and a refresh rate of 120 HZ. Participants were seated approximately 60 cm from the screen. Both facial stimuli were 9.8 degrees of visual angle (deg VA) high and 8.1 deg VA (measured from ear to ear) and presented in the center of the screen. The targets ‘L’, ‘T’ and the non target ‘X’ were 0.6 deg VA high and 0.6 deg VA wide and presented 5,5 deg VA from the facial stimuli on the midline of the screen.

### Procedure

The gaze cuing task was similar to the one employed in many previous studies [1], [9], [23]. Participants were instructed to focus their attention on the centrally presented fixation point that was visible for 500 ms. After the fixation cue, a non-dominant looking female face or a dominant looking male face appeared with a straight ahead gaze for 1000 ms. This face was subsequently replaced with exactly the same face, either with a gaze directed to the left, right or straight ahead (control condition, no change). The peripherally located target (‘L’ or ‘T’) appeared either at 200 or 800 ms (SOA) after the presentation of the second face, and its location was unrelated to the gaze direction of the face stimulus (see Figure 1 for an example of the trial sequence and a picture of the dominant looking male and non-dominant looking female faces used as gaze cue).

Participants were briefed about the random location of the target and instructed to ignore the face. The target appeared on the same height of the facial gaze and symmetrically with a non-target (‘X’) located on the opposite side of the face. We chose to use a balanced display, one in which two onsets were presented, such that the onset of the target would not additionally bias target selection as may have been the case with a single onset. When the target letter was presented opposite of the gaze direction, it constituted an invalid trial; a target letter congruent to the gaze direction constituted a valid trial. The participants were instructed to press the up (‘T’) and down (‘L’) arrow on the keyboard as accurately and immediately as possible when they saw the target letter. The response time (RT) is measured as the time between presentation of the target letter and pressing a response key. The gaze cuing effect is measured by subtracting the mean average RT of the valid trial from the mean average RT of the invalid trial.

The experiment consists of a repeated measures design with 4 within subject factors; Prime condition (threat/no threat), SOA (stimulus onset asynchrony): 200 and 800 ms, Face (non-dominant looking female and dominant looking male) and Validity (valid, invalid and control). Prior to the start of the experiment the participants practiced one block of trials (48 trials). An experimental block of the gaze cuing experiment consisted of a single presentation of the prime condition (20 threat or no-threat pictures) and followed by a block of 288 trials (6 blocks of 48 trials). Twenty-three participants started with the ‘threat’ condition and twenty-two participants started with the ‘no threat’ condition. The participants switched prime-condition after the first block of 288 trials in the gaze cuing experiment, such that prime-condition was counterbalanced between participants. Participants had a 5 minute break between the two blocks of the experiment. Thus, each observer completed 576 trials, divided in two blocks of 288 trials each with a separate prime-condition. Face type, SOA, location of target, gaze direction and target letter are fully randomized in every block sequence.

### Manipulation Checks

The participants filled in two questionnaires immediately after they finished the complete gaze cuing task. They started with rating the male and female face on 5 different face features (dominance, masculinity, leadership, trustworthiness and attractiveness) along a 7 point scale (1 = low degree, 7 = high degree). The questionnaire was presented on a printed form on which both the male and female face were displayed. Analysis of the face ratings showed that participants rated the male face as significantly more dominant, masculine, and leader-like compared to the female face (N = 45, *t*>9.47, *p*<.01). Conversely, the female face was rated significantly higher in trustworthiness and attractiveness than the male face (N = 45, *t>*5.02, *p*<.01) – see Table 1 for the mean ratings and standard deviations per feature.

**Table 1:** Mean Rating Scores and Standard Deviations of The Male and Female Face on Various Features (1 = high degree, 7 = low degree).

	Male face	Female face
**Dominance**	6.27 (1.16)	3.20 (.78)*
**Masculinity**	6.18 (1.57)	2.80 (1.32)*
**Leadership**	5.87 (1.08)	3.36 (1.38)*
**Attractiveness**	2.91 (1.41)	4.11 (1.37)*
**Trustworthiness**	2.98 (1.01)	5.53 (.76)*

Note. SD = between brackets (.), N = 45.

* means differ at p<.01.

After the face ratings, the pictures from the threat and no threat condition were displayed once more and participants filled in the PANAS questionnaire after they saw the pictures from each condition again. Analysis of the affect questionnaire, a short version of the PANAS (the Positive and Negative Affect Scale; [30]), showed that, after seeing the pictures in the threat-condition, compared to the no threat-condition, participants felt significantly less positive (*M = *2.10 vs. 3.51, *SD* = .70 and.71; N = 45, *t = *9.75, *p*<.01) and significantly more negative (*M* = 2.65 vs. 2.03; *SD* = .87 and.40; N = 45, *t = *4.94, *p*<.01). These results suggest that the Prime-manipulation was successful as it modulated emotional affect.

### Initial Processing of Data

Incorrect responses as well as trials in which the response time was greater than three standard deviations above or below the mean of the participant were excluded from the final analysis (fewer than 5% of cases).

## Results

We excluded participants who did not fill in the two questionnaires (N = 3) or had extreme outliers in their data that affected the normal distribution of our data (N = 1; this participant had an average gaze cuing effect of 219.9 ms in the ‘no threat’ condition for 800 ms [*M* condition = 15.67, *SD* condition = 32.7]).

The mean average reaction times in the gaze cuing task for the dominant looking male face, the non-dominant looking female face in each of two prime conditions and for each of the three validity–conditions are displayed in Table 2.

**Table 2:** Mean reaction time (in ms) and standard error of the mean for target discrimination in each of the different conditions.

Prime	No Threat condition											
SOA	200 ms						800 ms					
FACE	Male			Female			Male			Female		
Validity	I	V	C	I	V	C	I	V	C	I	V	C
**Mean**	566.8	557.2	562.3	568.2	553.6	562.3	560.0	534.8	556.2	553.0	542.5	551.3
**SEM**	13.6	12.2	13.3	13.7	13.0	15.2	14.4	12.8	14.9	14.2	14.3	13.2
**Prime**	**Threat condition**											
**SOA**	**200 ms**						**800 ms**					
**FACE**	**Male**			**Female**			**Male**			**Female**		
**Validity**	**I**	**V**	**C**	**I**	**V**	**C**	**I**	**V**	**C**	**I**	**V**	**C**
**Mean**	569.7	559.0	557.2	557.8	560.9	556.9	553.2	539.8	551.6	549.8	544.9	550.1
**SEM**	14.4	13.0	13.1	11.8	14.0	13.1	13.1	13.4	12.6	12.5	13.9	13.8

*Note.* I = Invalid, V = Valid, C = Control (no change), SEM = Standard Error of the Mean, N = 45.

**Figure 2:**
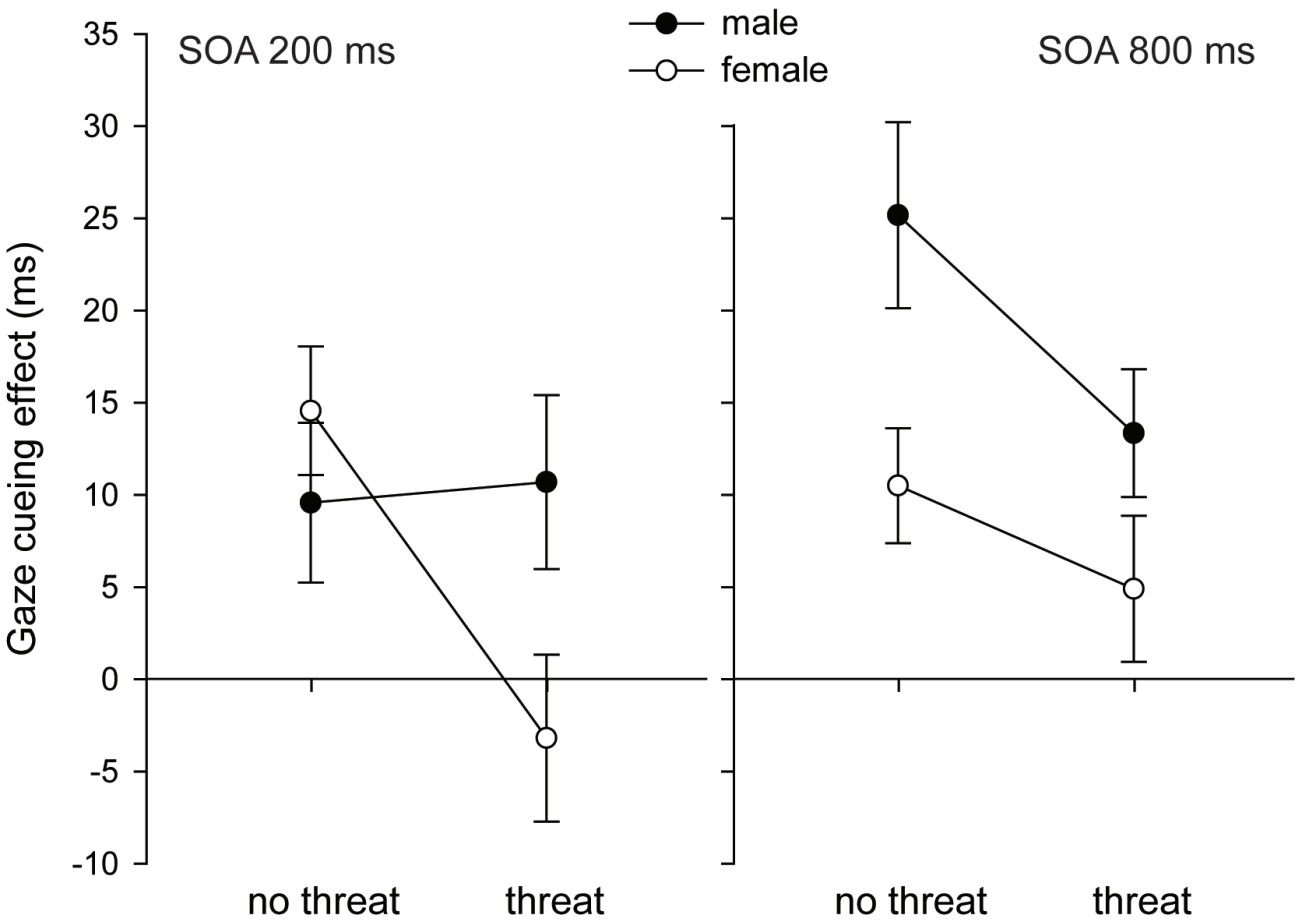
Threat and gender modulate gaze following. Significant interaction effect between: Prime condition, SOA and Face (*F*(1,44) = 4.36, *p* = .043), the error bars in the figure represent within-subject standard error of the mean.

The gaze cuing effects (RT_invalid – RT_valid) were analyzed using a repeated measures design ANOVA (*within subject factors*: Prime condition: threat/no threat, SOA: 200/800 ms, Face: female/male). Tests of within subjects effects revealed significant main effects for Face and Prime (*F*(1,44) = 5.99, *p = *.018, η_p_^2^ = .12; *F*(1,44) = 6.69, *p = *.013, η_p_^2^ = .13). Specifically, the gaze cue effect from the dominant male face was larger overall compared to the female face (14.70 ms versus 6.70 ms). Moreover, following the ‘no threat’ prime condition a significant larger gaze cuing effect was found compared to the ‘threat’ condition (14.95 ms versus 6.44 ms). The three-way interaction between Prime condition, SOA and Face also reached significance (*F*(1,44) = 4.36, *p = *.043, η_p_^2^ = .090, see Figure 2). No other significant effects were found.

A mixed ANOVA showed that there were no gender differences between participants in gaze cuing effects across the different conditions (all *F*<1.46, all *p*>.23). Although previous research has shown that female participants have larger gaze cuing effects compared to male subjects [31], we did not find any such differences in our study. We also checked if the order of the primes (Threat first vs. No threat first) influenced our results and ran a mixed repeated measures ANOVA with Prime/Face and SOA as within-subject factors and Order as a between-factor. Order did not have a main effect (*F*<1). Order significantly affected the interaction between Prime and SOA (*F*(1, 43) = 12.09, *p* = 0.001), but no other interactions were significant (all *p*’s>.1).

In the introduction we hypothesized that the difference in gaze cuing between the dominant male and non-dominant female faces would be particularly pronounced in a threatening emotional context. To investigate this directly, a planned contrast was performed to compare the gaze cue effect between the two faces in the threat condition only, collapsed over SOA. The results revealed a significant interaction (*F*(1, 44) = 5.93, *p* = 0.019, η_p_^2^ = 0.11), showing that the gaze cue effect for the male face was significantly larger than the gaze cue effect for the female face in the threat condition. A similar contrast in the no-threat condition was not significant (*F*(1, 44) = 1.53, *p* = 0.22, η_p_^2^ = 0.03).

For each condition, the gaze cuing effect was calculated and t-tests for single samples were conducted to test if gaze effects were significantly different from zero (no gaze cuing effect). The results reveal that in the threat condition the female face does not elicit a gaze cuing effect at 200 and 800 ms viewing time. In all other conditions the gaze cuing effects were significantly different from zero (see Table 3).

**Table 3:** Descriptive statistics of the gaze cue effect (RT invalid- RT valid in ms) and results of one-sample t-tests (tested against 0, no difference RT valid vs. invalid cue).

Prime	No threat prime condition				Threat prime condition			
SOA	200 ms		800 ms		200 ms		800 ms	
Face	Male	Female	Male	Female	Male	Female	Male	Female
Mean	9.57	14.56	25.17	10.50	10.69	−3.19	13.35	4.90
SEM	4.64	3.77	5.78	3.72	4.73	4.54	4.33	4.77
Sig.	**.045** *	**.0004** *	**.0001** *	**.0072** *	**.029** *	**.49**	**.0035** *	**.31**

*Note.* SEM = Standard Error of the Mean,

* *p*<.05, N = 45.

The errors participants made during the experiment were analyzed with a repeated measures ANOVA (within subject effects: Prime/SOA/Face). The error rates were calculated by subtracting the errors in the valid trial from the errors in the invalid trial (see Table 4). There were no significant differences in error rates between the different conditions (all *F’s*<2.04, all *p’s*>.16).

**Table 4:** Percentage of errors and standard error of the mean in each of the different conditions.

Prime	No Threat condition											
SOA	200 ms						800 ms					
FACE	Male			Female			Male			Female		
Validity	I	V	C	I	V	C	I	V	C	I	V	C
**% Errors**	2.69	2.31	2.31	2.22	3.06.	2.59	2.13	2.41	1.76	2.78	2.41	1.67
**SEM**	0.53	0.61	0.74	0.52	0.65	0.65	0.45	0.67	0.34	0.59	0.57	0.47
**Prime**	**Threat condition**											
**SOA**	**200 ms**						**800 ms**					
**FACE**	**Male**			**Female**			**Male**			**Female**		
**Validity**	**I**	**V**	**C**	**I**	**V**	**C**	**I**	**V**	**C**	**I**	**V**	**C**
**% Errors**	2.59	2.96	1.76	3.33	2.5	2.87	2.78	2.5	1.85	2.59	1.94	2.22
**SEM**	0.57	0.56	0.60	0.64	0.57	0.71	0.78	0.58	0.43	0.44	0.47	0.47

*Note.* I = Invalid, V = Valid, C = Control (no change), SEM = Standard Error of the Mean, N = 45.

## Discussion

The present study showed that facial cues interact with emotional context to modulate gaze cuing. Our findings revealed first that the overall gaze cue effect was larger for a dominant looking male face than a non-dominant looking female face. This is consistent with prior studies which have shown that gaze cuing effects are modulated by facial features signaling social attributes such as dominance [9], familiarity [23], status [15] and group membership [10]. Our findings extend these results, however, by showing that the emotional context critically interacts with these facial cues to modulate gaze cuing. Our study revealed that the gaze of a non-dominant female face did no longer cue attention in a threatening, dangerous context.

One explanation for why the gaze cue of the dominant looking male face was overall more effective than the non-dominant looking female face may be found in evolutionary psychology theories about face perception and leadership. Research suggests that different implicit leadership prototypes are activated in different environments, with a masculine looking leader being followed more often, and especially at times of danger or crisis [16], [20], [25]. This “think leader, think male” bias might be the result of an evolved, highly automatic decision rule to follow individuals who can offer safety and protection in dangerous environments such as what early humans must have faced during evolutionary history [22]. This idea lead us to hypothesize that potential differences between the dominant male and non-dominant female face in gaze cuing would be particularly pronounced in situations eliciting threat. Our findings showed that participants stopped following the female face gaze in a dangerous context. The present research might thus help to understand why prejudices against female leadership are very hard to eradicate, even in modern societies, and perhaps especially in times of crisis [32].

Our findings are, in two ways, surprising: First, the dominant male face produced an overall larger gaze cuing effect, despite this face having smaller eyes and visible sclera, which in theory could have made it more difficult to follow the male rather than the female gaze [33], [34]. Some people have suggested that the gaze cue effect is determined in part by low-level differences between gaze-conditions [33]–[35]. Widening of the eye may facilitate the perception of this contrast and the subsequent movement of the eyes toward the target location, thus potentially enhancing the orientation effect. However, the present results are at odds with these suggestions. Thus we may tentatively conclude that the processing of the male and female faces is predominantly influenced by higher-level social and contextual effects.

Second, the male face was rated by participants as significantly lower in trustworthiness and attractiveness than the female face. Intuitively, high ratings on attractiveness and trustworthiness – two qualities that are often associated with leadership [32] – should benefit gaze cuing. That is, these traits should have acted in favor of the female face cuing. The present results suggest that facial dominance may trump facial attractiveness and trustworthiness, especially when individuals are confronted with danger. It may prove insightful in future studies to investigate the potential contribution of facial cues of attractiveness or trustworthiness in settings that elicit either mating or collaboration goals. A weakness of this study is that facial cues of gender were confounded in our study with cues of psychological dominance and masculinity or femininity. A challenge for future research will be to disentangle the influence of gender from dominance or masculinity in face cues, and investigate the separate effects as a function of emotional context.

The current findings provide insight into why some studies have found effects of facial features on gaze cuing, whereas others have not. Tipples showed that the gaze cue effect for fearful faces is larger than for neutral faces [36], and Graham found that emotional expressions in faces – happy and especially fearful – elicit cuing effects yet at longer viewing times only [37]. However various other studies have found no modulation effects of facial expression in gaze cuing [38]–[40]. This could be potentially explained by differences in activated emotional context. In the study by Tipples the gaze cue effect for fearful faces was modulated by the emotional state of the participant (especially at the short SOA). A significant correlation was found in that particular study between the personality trait fearfulness and gaze cuing such that higher degrees of fearfulness among participants predicted their orienting to the eye gaze for fearful faces. The present findings are similar because they suggest that gaze cuing is stronger if there is a ‘match’ between the facial cues and the emotional context. Our finding is also consistent with previously reported congruency effects between the emotional expression of the face cue and the response target if this target carries emotional valence [41]–[44].

### Conclusion

Our study is to our knowledge the first to show that certain features of a cued face are influential only in a given emotional context. By showing that individuals do not follow the gaze of a non-dominant looking female face in a threatening environment, this research provides novel insights into the gaze cuing literature as it fits nicely with a more ecological approach [45] as well as the evolutionary psychological literature on face perception, social coordination, and leadership [22].
